# Assessing Nurses’ Knowledge and Attitudes Towards Biosimilars: Results from a National Survey

**DOI:** 10.3390/healthcare14040524

**Published:** 2026-02-19

**Authors:** Silvia Domínguez-Fernández, Susana Montenegro-Méndez, Macarena González-Rodríguez, Noelia Cano-Sanz, Ana María Duro-Martínez, Pablo Bella-Castillo, Guadalupe Fontán-Vinagre, Roberto Guerrero-Menéndez

**Affiliations:** 1 Villa de Vallecas Community Health Center, C. Sierra de Gádor 68, 28031 Madrid, Spain; sildom01@ucm.es; 2 Spanish Institute of Nursing Research, C. Sierra de Pajarejo 13, 28023 Madrid, Spaing.fontan@ieinstituto.es (G.F.-V.); 3 UCM Research Group “Public Health-Lifestyles, Nursing Methodology and Care in the Community Environment” Department of Nursing, Faculty of Nursing Physiotherapy and Podiatry, Complutense University of Madrid, 28040 Madrid, Spain; 4 Inflammatory Bowel Disease Unit, Hospital Universitario Puerta de Hierro Majadahonda, C. Joaquín Rodrigo 1, 28222 Majadahonda, Spain; mgonzalezrodriguez3@salud.madrid.org (M.G.-R.); pbella@idiphim.org (P.B.-C.); 5 Inflammatory Bowel Disease Unit, Hospital Universitario de León, C. Altos de Nava s/n, 24071 León, Spain; ncanosa@saludcastillayleon.es; 6 Pharmacy Unit, Inflammatory Bowel Disease Unit, Hospital Universitario Fundación Alcorcón, C. Budapest 1, 28922 Alcorcón, Spain; anamaria.duro@salud.madrid.org

**Keywords:** knowledge, biosimilars, interchangeable products, nurses, survey

## Abstract

**Highlights:**

**What are the main findings?**

- Nurses in Spain generally demonstrate only basic knowledge of biosimilars. Higher knowledge levels are more common among those who have completed industry-led training and/or work in units where biosimilars are already in use.
- Working in units where biosimilars are routinely administered or prescribed increases nurses’ confidence in their safety and efficacy. This confidence also rises progressively with higher levels of biosimilar knowledge.

**What are the implications of the main findings?**

- There is a substantial opportunity to improve nurses’ knowledge of biosimilars in European countries, at a moment when they are willing to learn and recognize the benefits these therapies offer to patients and healthcare systems.
- Strengthening education should begin with integrating biosimilar content into academic curricula and expanding diverse training modalities in collaboration with the pharmaceutical industry.

**Abstract:**

**Background/Objectives:** Nurses play a key role in supporting patient adherence to biosimilars, which requires adequate knowledge of biological therapies. This study aimed to assess nurses’ knowledge and attitudes toward biosimilars in Spain, representing the first nationwide assessment of Spanish nurses’ knowledge and predictive determinants of reliance on biosimilars. **Methods:** A self-administered, web-based survey was distributed between May and June 2024. Associations were explored using Spearman’s correlation and Fisher’s exact tests, and predictors were examined with ordinal regression models. **Results:** A total of 402 nurses responded. Most (63.7%) reported at least basic knowledge of biosimilars. Access to industry-led training was strongly associated with higher knowledge (OR = 11.256; *p* < 0.001), while lack of awareness of workplace biosimilar use was linked to lower knowledge (OR = 0.176; *p* < 0.001). Confidence in biosimilar safety and efficacy increased with knowledge level (ORs 3.823–14.594; all *p* < 0.001) and was higher among nurses working with biosimilars in their units (OR = 3.959; *p* = 0.004) and in hospital ambulatory care services (OR = 2.506; *p* = 0.022). **Conclusions:** Spanish nurses predominantly demonstrate basic knowledge of biosimilars, highlighting the need for broader training access. Industry-led training was the strongest modifiable factor to improve knowledge and confidence. Strengthening collaboration with the pharmaceutical industry may support informed practice and enhance patient adherence to biosimilar therapy.

## 1. Introduction

The use of biological medicines has markedly improved the treatment of numerous diseases [1]. However, their increasing utilization (driven by the growing burden of chronic conditions and their high costs) has led health systems to promote the development and prescription of biosimilars [2].

Biosimilars are medicines that are highly similar to their reference biologic products in terms of structure, quality, safety, efficacy, and immunogenicity [3]. Since the first biosimilar was approved in 2006, the European Commission has authorized 25 active substances across 131 medicines [4]. Approved biosimilars are considered scientifically interchangeable with their corresponding reference medicines. Clinical interchangeability between two biosimilars derived from the same reference product may also be possible [5]. Importantly, biosimilars are not equivalent to generic medicines, as their complex and heterogeneous manufacturing processes cannot produce molecules identical to the reference biologic [6].

The growing availability of biosimilars can enhance patient access to biological therapies and alleviate the economic burden on health systems, owing to their lower cost compared with reference medicines [7,8]. The introduction of biosimilars has led to price reductions in several European countries with the main adoption at the hospital level [9]. In Spain, the use of biosimilars has led to a 28% reduction in costs compared to reference medicines, accounting for 7.7% (€981 million) of total pharmaceutical expenditure [10].

Although biosimilars are relatively new to clinical practice, they are often subject to heightened regulatory and clinical vigilance, which may initially lead to increased reporting of immunogenicity related reactions or injection site events compared with their reference biologics [11]. However, current evidence consistently shows that biosimilars and their reference products demonstrate comparable safety profiles and similar rates of immunogenicity [12,13].

Despite the proven safety and effectiveness of biosimilars, patient misconceptions regarding quality and efficacy still compromise therapeutic adherence and clinical outcomes [14]. To address this challenge, multi-stakeholder approaches should involve physicians, pharmacists and nurses [15]. Nurses play a pivotal role in medication management, pharmacovigilance, and patient education regarding reference medicines and biosimilars, promoting understanding of interchangeability and thereby reducing the nocebo effect [16,17].

While most research has examined the knowledge and attitudes of physicians and pharmacists toward biosimilars [18,19,20,21], evidence on nurses’ experiences remains limited [22]. Studies involving nurses have primarily examined knowledge and attitudes toward biosimilar use, with particular emphasis on reliance in their safety and efficacy as a key attitudinal component [23,24]. Friganović et al. [25] conducted an international survey and identified concerns about conflicting attitudes and knowledge gaps among nurses, which were recognized as threat to medication safety, patient trust and switching implementation [26]. Other studies have explored nurses’ perspectives on barriers and facilitators to biosimilar implementation [15,22,27]. Nurses rely on diverse learning sources and platforms for information on biosimilars, and access to these resources may influence their knowledge and attitudes [25,28]. Moreover, nurses’ knowledge and attitudinal characteristics (such as confidence in biosimilars) appear to be shaped by sociodemographic and professional factors [22,25]. However, none of these associations have yet been studied within the Spanish healthcare context.

To address these gaps, the present study provides the first nationwide assessment of Spanish nurses’ knowledge, attitudes, and perceptions regarding biosimilars. Specifically, it examines the following research questions (RQs):

RQ1: What levels of knowledge, attitudes, and perceptions toward biosimilars are held by nurses working in Spain?

RQ2: How are nurses’ knowledge and attitudes toward biosimilars associated with sociodemographic factors (e.g., age, sex, years of experience, region) and professional characteristics (e.g., clinical setting, prior exposure to biosimilars, access to training)?

RQ3: Which sociodemographic and professional variables predict nurses’ knowledge of and reliance on biosimilars?

Beyond providing descriptive data, this study contributes to the existing literature by examining the sociodemographic and professional determinants of nurses’ knowledge and reliance on biosimilars within a national healthcare system characterized by significant biosimilar uptake. By integrating descriptive and predictive analyses, the study advances understanding of the structural and educational factors that may influence biosimilar implementation in clinical practice.

## 2. Materials and Methods

### 2.1. Study Design

This study was a self-administered, web-based survey designed to assess nurses’ knowledge and attitudes toward biosimilars in Spain. It employed a cross-sectional, quantitative, descriptive and correlational design. The reporting of this study conforms to the statement “Strengthening the reporting of observational studies in epidemiology” (STROBE) [29], in conjunction with the Consensus-Based Checklist for Reporting of Survey Studies (CROSS) [30].

### 2.2. Questionnaire Details

The survey tool was developed in four stages. First, a comprehensive literature review and assessment of existing instruments were conducted. The questionnaire used was based on the survey developed by Friganović et al. for use in a study involving 11 European countries [25]. In the second stage, the items were conceptually adapted and rewritten directly in Spanish to ensure alignment with Spanish nursing terminology and practice. As the original instrument consisted mainly of factual demographic questions, single item measures of knowledge, and categorical attitude items, a full cross cultural adaptation procedure (including translation and back translation) was not required. The adapted version was reviewed by experts and piloted among practising nurses to ensure clarity and contextual suitability. The expert panel comprised five nurses with extensive clinical experience in the use of biosimilars. Three of these experts were selected from among the authors of the Guide to Biosimilar Medicines for Nurses [31], and they, in turn, identified two additional experts who had more than five years of clinical experience working with biosimilars. Based on the feedback obtained from this panel, the survey underwent further refinement during the third stage.

In stage four, a pilot survey was conducted to confirm feasibility in a small sample of nurses (*n* = 40). The pilot responses were included in the final sample and were also used, together with feedback from the expert panel, to confirm the content validity of the questionnaire. The survey tool is provided in Supplementary Table S1.

A 23-item self-administered questionnaire was developed to collect data, comprising single-choice, multiple-choice, and open-ended questions. General participant information included sociodemographic variables such as age, sex, region of work, years of professional experience, clinical setting (with two sub-questions on hospital areas and other areas), business sector, and postgraduate qualifications. Specific items assessed nurses’ knowledge of biosimilars (e.g., self-assessed level of knowledge, definition of biosimilar, distinction between biosimilars and generics, and examples of known biosimilars), professional factors (e.g., the use of biosimilars in the workplace and access to training courses on biosimilars) and reliance toward biosimilars (e.g., reliance on biosimilars’ safety and efficacy, willingness to receive training, perceived barriers and facilitators, perceived benefits, and nurses’ role in biosimilar education). Additional questions about nurses’ perception were included for descriptive purposes.

For modelling and to ensure adequate cell counts, several categorical variables were aggregated a priori. Autonomous Communities were grouped into four macro-regions (North, Midlands, East, South); hospital units were collapsed into Ambulatory Services (day hospital/outpatient clinics) versus specific units (inpatient, pharmacy, ICU, other); work sector was reduced to Public versus Private/Semi-private; postgraduate training was recoded to reflect the highest award level (No certified training, Expert certificate, MSc./Nurse Specialist, PhD); and confidence in biosimilars was collapsed into three levels (I don’t know/No/Low confidence, Average, Much/Total confidence). Full mappings of original to aggregated categories are provided in Supplementary Table S2.

### 2.3. Sample Characteristics

According to Zamora-Romero et al. [32], 296,462 nurses were employed in the Spanish healthcare system in 2022. The study used non-probabilistic convenience sampling and included registered nurses providing direct patient care in Spanish healthcare settings. Nurses working in non-clinical areas or on leave were excluded. Based on a 95% confidence level, a 0.05 margin of error, and an assumed proportion of 0.5, the required sample size was calculated as 380 respondents.

### 2.4. Survey Administration

The survey was administered via the EU Survey online platform (EU Public License, European Commission; https://ec.europa.eu/eusurvey (accessed on 10 June 2025), which supported participation from mobile devices. According to the pre-test performed, completion time was estimated at 10–15 min, although no time limit was imposed. All items in the questionnaire were mandatory. To prevent automated submissions, the platform required participants to complete a CAPTCHA challenge before submitting their responses. Additionally, each IP address was restricted to a single submission. The survey interface displayed a cover text outlining the purpose and importance of the study for nursing development and patient safety, emphasizing the absence of right or wrong answers and ensuring anonymity, confidentiality, and data privacy.

The survey link was disseminated through several professional channels, including email lists, institutional and professional mailing lists, and the communication platforms of national and regional nursing societies (including LinkedIn and the official WhatsApp channel of the Spanish Institute of Nursing Research). The survey remained open for responses from May to June 2024. Due to the nature of these distribution channels, it is not possible to determine the exact number of nurses who received the invitation. Although a precise response rate cannot be calculated, transparent reporting of the distribution process and its limitations is recommended in survey research [33].

Respondent-related and measurement-related sources of common method bias (CMB) were addressed through procedural measures, including survey design strategies (e.g., clear instructions, assurance of anonymity, and avoidance of ambiguous items) and methodological separation (e.g., use of varied item formats and distinct measures for independent and dependent variables) [34].

### 2.5. Ethical Considerations

The study was conducted in accordance with the Declaration of Helsinki [35], and the protocol was approved by the Ethics Committee of the Príncipe de Asturias University Hospital in Madrid on 19 March 2024 (Approval No. 08/2024). Participation in the survey implied informed consent, and respondents were provided with information regarding the study’s purpose, confidentiality, and data privacy.

### 2.6. Statistical Analysis

Data were exported from the EU Survey platform into Jamovi (Version 2.5; The jamovi project, 2025; https://www.jamovi.org (accessed on 10 June 2025)) [36] for analysis. Descriptive statistics (frequencies, percentages, means, and standard deviations) were calculated for continuous, ordinal, and nominal variables. Concordance between nurses’ self-reported knowledge and objective measures (correct definition, differences between generics and biosimilars, and number of biosimilars identified) was assessed using Kendall’s *W* coefficient (poor: <0.00; slight: 0.00–0.20; fair: 0.21–0.40; moderate: 0.41–0.60; substantial: 0.61–0.80; and almost perfect: 0.81–1.00) [37]. Correlations of nurses’ knowledge and reliance with ordinal variables were examined using Spearman’s rank correlation. Associations of nurses’ knowledge and reliance with nominal variables were tested using Fisher’s exact test due to small cell counts expected (<5). Cramer’s *V* was used to measure the strength of association (weak: >0.05; moderate: >0.10; strong: >0.15; and very strong: >0.25) [38]. Variables showing statistical significance were included in ordinal regression models to identify factors predicting nurses’ knowledge and reliance on safety and efficacy toward biosimilars. A *p*-value < 0.05 was considered statistically significant for all analyses.

## 3. Results

The survey was distributed to 402 nurses across the country. Since all fields were mandatory for submission, all questionnaires were valid.

### 3.1. Sample Demographics

The distribution of sample demographics and their representativeness compared to available data on registered nurses in Spanish healthcare [32,39] are presented in Table 1. The most represented age group was 41–65 years (70.4%). Female participants comprised 84.8%, and the most frequent work region was the Community of Madrid (30.1%). Most participants were employed in the public sector (83.6%), and the majority worked in hospital-based care settings (69.2%). Regarding postgraduate education, most had completed continuing education courses (68.7%), while only 3.0% had earned a PhD.

### 3.2. Responses to Nurses’ Knowledge, Perception and Attitudes Toward Biosimilars

In the survey, 63.7% of participants reported having at least basic knowledge of biosimilars, while only 6.0% indicated advanced knowledge. The correct EMA definition of biosimilars (“a biological medicine that has lost its patent and is equivalent in efficacy and safety to its reference medicine”) [40] was selected by 46.5% of respondents. Additionally, 66.9% correctly identified the differences between biosimilars and generic medicines. The most recognized marketed biosimilars included Enoxaparin sodium (56.2%), followed by insulin glargine (48.5%) and rituximab (36.3%).

Among respondents, 89.3% expressed willingness to receive training, with the workplace being the preferred source of information (43.8%). However, only 14.2% reported having access to work-based training, and just 9.0% of those indicated that it was delivered by the pharmaceutical industry. Regarding the use of biosimilars in the workplace, 63.9% responded affirmatively, while 26.9% were unsure. The main perceived benefit of biosimilars was cost reduction (52.9%), whereas only 22.6% fully trusted their efficacy and safety. The most significant barrier to the safe use of biosimilars was insufficient knowledge and experience (68.7%).

The roles of healthcare professionals and nurses in the use of biosimilars were primarily related to patient education, administration, and promotion of therapeutic adherence, as reported by 58.7% of respondents. According to 65.4% of participants, all members of the multidisciplinary team share responsibility for providing patient information. Specific responsibilities were perceived as falling on pharmacists (46.3%), nurses (42.3%), and physicians (42.0%). Most participants (77.4%) believed that informing nurses would be the first step toward improving trust in the efficacy and safety of biosimilars. Complete answers to questions regarding knowledge, attitudes and perceptions are available in Supplementary Table S3.

### 3.3. Concordance of Nurses’ Self-Reported Knowledge with Objective Measures

Responses to self-reported knowledge demonstrated substantial agreement with objective measures, including correctly defining biosimilars, identifying biosimilars, and distinguishing between generic and biosimilar medicines (Kendall’s *W* = 0.648; *χ*^2^ (401) = 782; *p* < 0.001).

### 3.4. Nurses’ Knowledge and Reliance on Biosimilars vs. Sociodemographic and Professional Factors

After grouping categories, the results of multivariate tests indicate that nurses’ knowledge of biosimilars was weakly correlated with age (*r*_s_ = 0.111; *p* = 0.027). Table 2 shows strong associations in bivariate tests between knowledge and hospital units, as well as very strong associations with access to training courses and the use of biosimilars in the workplace.

Nurses’ reliance on biosimilars showed a moderate positive correlation with self-reported knowledge (*r*_s_ = 0.521; *p* < 0.001) and weak negative correlations with years of experience (*r*_s_ = −0.112; *p* = 0.024) and age (*r*_s_ = −0.116; *p* = 0.020). In addition, reliance was very strongly associated with the use of biosimilars in the workplace (*V* = 0.382; *p* < 0.001) and strongly associated with hospital unit (*V* = 0.213; *p* = 0.002).

### 3.5. Factors Predicting Nurses’ Knowledge and Reliance on Biosimilars

Table 3 shows that access to work-based training delivered by the pharmaceutical industry and awareness of biosimilar use in the workplace were the strongest predictors of higher levels of nurses’ knowledge about biosimilars. Furthermore, employment in ambulatory services (such as day-care units or outpatient clinics) demonstrated greater predictive strength compared with inpatient wards, ICUs or hospital pharmacy.

Nurses’ reliance on biosimilars increased progressively with higher levels of self-assessed knowledge. Confidence was also higher among nurses who used biosimilars in their workplace and among those working in ambulatory services or other hospital units, compared with those in inpatient settings. Neither age nor professional experience demonstrated a clear independent effect; however, professional experience acted as a moderator in the relationship between age and confidence. The results of the ordinal regression analysis are presented in Table 4.

## 4. Discussion

This study represents the first nationwide survey of nurses on this topic and provides insight into their knowledge, attitudes, and perceptions of biosimilars, as well as the training resources available to them. It also identifies several sociodemographic and professional predictors of nurses’ knowledge and their reliance on the safety and efficacy of biosimilars.

Most participants reported having only basic knowledge. However, the likelihood of higher knowledge levels was greater among nurses working in hospital ambulatory services, in settings where biosimilars are routinely used, and in contexts where training has been delivered by the pharmaceutical industry. Although age appeared to be associated with both knowledge and reliance, it was not identified as a significant predictor, underscoring the need to strengthen biosimilar-related training for nurses across all age groups. The knowledge-related findings of this study are consistent with those reported in other European countries [25], although their results suggested that age, gender, and educational level are associated factors. This discrepancy may be explained by differences in the representativeness of the populations studied. In our case, the sample closely reflected the available demographic data [32]. Previous studies have reported that most nurses exhibit low levels of knowledge about biosimilars, particularly when compared with physicians and pharmacists [23,24,26].

The higher knowledge levels observed among nurses working in ambulatory care services likely reflect sustained exposure to biologic and biosimilar therapies in medical and oncology daycare units. This exposure contributes to what Li et al. [41] referred to as “clinical specialized knowledge”, which encompasses, but is not limited to, understanding of the disease, drug administration and use, nursing and therapeutic procedures, and specific precautions related to biologic therapies. Clinically, this suggests that routine contact with biosimilars may reinforce experiential learning, supporting the integration of biosimilar-specific competencies into daily nursing practice. Extending similar exposure opportunities or simulation-based training to inpatient and primary care settings may help reduce observed knowledge gaps.

Limited awareness of biosimilar use within the workplace appears to be a substantial barrier to knowledge development, suggesting that organizational communication plays a critical role in shaping nurses’ competence. This challenge has been reported previously among nurses in the United Kingdom [23], where one in three respondents did not recognize that biosimilars were included in their local formulary, results that were poorer than those observed among physicians and pharmacists in the same study. Similarly, Barbier et al. [42] identified the absence of practical, context-specific information on biosimilars as a major barrier preventing healthcare professionals from fully understanding their clinical use. From a practice standpoint, this highlights the need for clearer institutional strategies (such as formulary visibility, structured onboarding, and interdisciplinary briefings) to ensure nurses are adequately informed about biosimilar implementation.

Self-reported knowledge was consistent with several objective indicators, including the accurate definition of a biosimilar, understanding of the differences between generics and biosimilars, and the number of biosimilars correctly identified. The accuracy rate for defining a biosimilar among Spanish nurses was lower than the 64.23% reported among Chinese nurses [24], but higher than the 25.8% observed in the Austrian study by Binder and Zeitlinger [26]. When compared with other healthcare professionals in Spain, nurses’ accuracy was similar to the 42% identified among primary care physicians [20], whereas the knowledge gap appears to be smaller among pharmacists and specialized hospital physicians [18]. In line with this pattern, a survey conducted in the United States showed that pharmacists demonstrated a more accurate understanding of biosimilars than physicians, largely due to their greater familiarity with the relevant regulatory frameworks and dispensing processes [43].

Understanding the differences between generics and biosimilars was reported to be lower among nurses than among physicians in Spain [20], while remaining comparable to that observed among nurses in other European countries [25], as well as among physicians and pharmacists in similar studies [21]. This distinction is critical, as a clear understanding of how generics differ from biosimilars is essential for ensuring consistency in patient education and for fostering trust among patients and their families [44].

Among the biosimilars currently used in the Spanish clinical context, only four could be considered relatively well known by nurses. The remaining agents were substantially less familiar, and even the recognition rates for the most widely used biosimilars were unexpectedly low. One possible explanation for these findings is the frequent use of commercial brand names in routine clinical practice, which may hinder nurses’ ability to correctly identify the specific biosimilar prescribed [45]. Mico-Pérez et al. [20] similarly reported low familiarity with biosimilars, noting that six out of ten primary care physicians were unable to mention any marketed biosimilar, likely reflecting entrenched prescribing habits centred on reference biologics. In contrast, Barbier et al. [46] found a more robust integration of biosimilars into clinical practice in their international survey, with more than half of respondents indicating that they regularly prescribed the biosimilars listed. These contrasting findings may reflect differences in professional roles, prescribing responsibilities, exposure to biosimilars, and national implementation strategies.

The high willingness to receive training, particularly among nurses with lower baseline knowledge, represents a critical opportunity for intervention. Only a small proportion reported having received any information about biosimilars during their undergraduate studies, highlighting important opportunities to strengthen most nursing bachelor’s programs. These findings support the integration of biosimilar-related content into undergraduate nursing curricula [47]. Clinically, this readiness may facilitate the successful implementation of educational initiatives aimed at improving medication safety, patient education, and adherence, especially during biosimilar initiation or switching.

In relation to training opportunities within the clinical environment, medical and oncology ambulatory care services are among the settings where pharmaceutical industry representatives are more likely to deliver educational sessions. Access to this type of training has been shown to enhance nurses’ knowledge and, subsequently, to increase reliance on key aspects such as the effectiveness, safety, and cost-effectiveness of biosimilars; therefore, such training should ideally be provided before biosimilars are introduced into clinical practice [48]. Consistent with our observations, participation in biosimilar-related continuous training remains suboptimal, with only 65.7% of pharmacists and 50.7% of physicians reporting relevant CE credits [43]. Inequities in access to continuing professional development have been reported in other healthcare contexts, where disparities between urban and rural settings, as well as between hospital and non-hospital environments, have been identified [49].

Most nurses in Spain expressed a preference for remote training modalities (including online courses, guides, and webinars) mirroring trends reported among nurses in other European countries [25,28]. Although the use of online training has expanded substantially since the COVID-19 pandemic, the evidence indicates no significant differences in effectiveness between delivery modes (face-to-face, online, or blended). Instead, training approaches should be adapted to the specific educational context and learners’ needs [50].

The observed association between prior training and greater confidence in the safety and efficacy of biosimilars suggests that structured educational exposure may play a key role in shaping clinicians’ perceptions. As previously mentioned, younger age does not influence on reliance, although previous studies have shown that newly graduated nurses typically demonstrate medication-management competence at an advanced-beginner level [51,52,53]. This suggests that targeted training and early clinical exposure may help bridge initial gaps in confidence and support more consistent reliance on biosimilars across experience levels.

The proportion of respondents who were able to identify the benefits of using biosimilars and recognize the nurse’s role in patient education was consistent with their overall level of confidence in the safety and efficacy of these medicines. However, some concerns emerged regarding perceived limitations when administering a biosimilar without providing adequate patient information. Educating patients and involving them in shared decision-making are essential strategies to foster trust in biosimilars [44]. Nurses working in ambulatory and community settings are particularly well positioned to support this process, with the potential to enhance clinical outcomes across a wide range of conditions [1]. Willingness to recommend biosimilars also varies by profession, with pharmacists more likely than physicians to recommend a biosimilar to treatment-naïve patients, paralleling our gradients in confidence [43].

Moreover, growing awareness of the financial implications of treatment decisions reflects a broader shift toward economic and quality-driven decision-making in healthcare compared with two decades ago [54]. This can be interpreted as a potential barrier, as international evidence shows that payer coverage and formulary placement (51.0%) and patient cost (41.0%) are the barriers most frequently rated as ‘extremely significant’, which aligns with the obstacles observed in our study [43].

Nonetheless, nurses are not viewed as isolated agents in this process; rather, nurses’ emphasis on shared responsibility within multidisciplinary teams reflects their recognition of biosimilar education as a collective clinical task rather than an isolated professional role. These findings align with previous reports in which nurses also assumed pivotal roles within interdisciplinary teams [46,55]. This perspective supports the development of coordinated educational strategies that align nurses, pharmacists, and physicians, thereby promoting consistent messaging and strengthening patient trust in biosimilar therapies. Overall, a multistakeholder approach (encompassing healthcare professionals, scientific societies, regulatory bodies, and patient associations) is essential to ensure effective patient education on biosimilars [16].

### 4.1. Strengths and Limitations of Research

The study’s national scope, broad sample, and diverse clinical settings enhance its generalizability and offer a comprehensive overview of nurses’ knowledge and perceptions of biosimilars. Limitations related to convenience sampling, self-report data, and single-item measures are acknowledged, with implications for future research and practice discussed.

However, this study has several limitations. Firstly, the survey was disseminated using non probabilistic convenience sampling through professional networks, which may have introduced self-selection bias and limits the generalisability of the findings to the wider nursing population in Spain. Secondly, because the survey was distributed across multiple mailing lists and society communication channels, it was not possible to determine the number of individuals who received or viewed the invitation; therefore, a traditional response rate could not be reliably calculated. This is a recognised limitation of online surveys without a defined sampling frame. Lastly, as the questionnaire comprised predominantly single item categorical measures, it was not possible to estimate internal consistency reliability indices. Although procedural measures were implemented to mitigate common method bias (CMB), statistical techniques for its assessment could not be applied because they typically require continuous or normally distributed indicators [56]. In our study, most questionnaire items were nominal, which would have required specialized adaptations and analytical tools that were not accessible to the research team at the time of analysis.

Another limitation is that, although the questionnaire was based on an instrument used previously in 11 European countries, a formal cross-cultural adaptation process was not undertaken. The items were adapted conceptually and rewritten in Spanish rather than translated using a structured linguistic validation protocol. While content was reviewed by experts and piloted for clarity, the lack of a full cultural adaptation may limit direct comparability with studies using the original version.

Consequently, conclusions regarding the strength of the observed relationships should be interpreted with caution. Future research employing probability sampling and multi-item scales would enhance representativeness and allow for a more comprehensive assessment of reliability measures.

### 4.2. Implications for Practice

Despite these limitations, the findings have important implications for nursing practice and healthcare services.

In general, our study indicates a positive trend toward enhancing nurses’ knowledge and attitudes regarding biosimilars. This is reflected in their willingness to develop greater competence in the use of these therapies, while at the same time acknowledging persistent gaps in access to adequate training—an issue also reported by Oqal et al. [57]. Addressing these gaps through the implementation of standardized educational resources could not only strengthen professional competence but also improve communication with patients during biosimilar switching processes, as demonstrated with the digital platform developed by Marras et al. [58].

The findings from this study may be applied to broader research contexts focused on the safe use of biosimilars and on understanding the drivers needed to implement actions that promote equitable and sustainable adoption of newly developed biological therapies in healthcare settings.

## 5. Conclusions

This national survey provides an overview of nurses’ knowledge of, and confidence in, biosimilars in Spain and identifies several demographic and professional factors that are associated with these outcomes. The findings suggest that working in clinical settings where biosimilars are routinely used, employment in hospital ambulatory care services, and exposure to training (particularly industry-led) are linked to higher levels of knowledge and greater confidence in the safety and efficacy of biosimilars.

While the findings highlight important patterns (particularly regarding training exposure and clinical context) the cross-sectional design and use of self-reported data preclude causal interpretation. Consequently, the results should be viewed as indicative rather than definitive. Despite these limitations, the results point to training as a potentially important and modifiable factor that may support nurses’ confidence and preparedness when involved in biosimilar administration, patient education, and adherence support.

Given nurses’ central role within interdisciplinary teams (particularly in medication management and patient education) national and European multistakeholder action plans should prioritize equal access to training opportunities. Such initiatives should begin with the integration of biosimilar-related content into academic curricula and the adaptation of diverse training modalities in collaboration with the pharmaceutical industry. Future research should explore how nurses’ competence in biosimilars influences patient education, acceptance, and therapeutic adherence, thereby informing strategies to improve the safe and effective use of biosimilars.

## Figures and Tables

**Table 1:** Distribution of sample demographics with representativeness compared to available data for registered nurses in Spanish healthcare.

Demographic Data (n = 402)	Frequency (n)	Percent (%)	Frequency (N) ^1^	Percent (%) ^1^
AGE (years)				
22–30	35	8.7	42,690	14.4 ^2^
31–40	78	19.4	77,080	26.0 ^2^
41–50	147	36.6	80,637	27.2 ^2^
51–65	136	33.8	64,036	21.6 ^2^
≥66	6	1.5	14,823	0.5 ^2^
SEX				
Female	341	84.8	249,621	84.2
Male	61	15.2	46,841	15.8
REGION OF WORK				
Andalusia	32	8.0	45,279	15.3
Aragon	36	9.0	11,046	3.7
Asturias	5	1.2	7679	2.6
Balearic Islands	2	0.5	7721	2.6
Canary Islands	8	2.0	15,026	5.1
Cantabria	2	0.5	4027	1.4
Castilla y León	44	10.9	16,892	5.7
Castilla-La Mancha	16	4.0	13,585	4.6
Catalonia	34	8.5	49,680	16.8
Region of Valencia	11	2.7	28,054	9.5
Extremadura	5	1.2	7398	2.5
Galicia	15	3.7	16,588	5.6
Community of Madrid	121	30.1	37,316	12.6
Region of Murcia	36	9.0	9938	3.4
Autonomous Community of Navarre	16	4.0	5339	1.8
Basque Country	15	3.7	17,665	6.0
La Rioja	4	1.0	2198	0.7
Ceuta y Melilla	0	0.0	1031	0.3
PROFESSIONAL ESPERIENCE (Min = 1; Max = 45).			*μ* ^3^ = 22.1	SD ^4^ = 10.4
CARE SETTING				
Hospital Care ^5^	278	69.2	201,406	67.9
Primary Care	71	17.7	42,094	14.1
Others ^6^	53	13.2	53,123	18.0
5. HOSPITAL AREAS				
Daycare Unit	21	7.9		
Intensive Care	31	11.7		
Inpatient unit	90	33.8		
Pharmacy Unit	6	2.3		
Outpatient Clinics	25	9.3		
Other	93	35.0		
6. OTHER (AREAS)				
Social and health centre	19	35.8		
Out-of-hospital emergencies	11	20.8	3959	7.45
Mutual insurance/occupational health companies	8	15.1		
Other	15	28.3		
BUSINESS SECTOR				
Semi-private	32	8.0	25,190	8.5
Private	34	8.5	73,207	24.7
Public	336	83.6	198,065	66.8
POSTGRADUATE EDUCATION				
PhD.	12	2.9		
MSc./Nurse Specialist	189	47.0		
Expert Certificate	80	19.9		
Continuous training courses/No postgraduate education	124	30.9		

^1^ Data from the reference population of nurses in Spain (*N* = 296,462) [28,34]. ^2^ Age groups in the study sample follow a different distribution compared with the reference population: <30 years, 30–39 years, 40–49 years, 50–59 years, 60–69 years, >69 years. ^3^ *μ* = Mean; ^4^ SD = Standard Deviation. ^5^ Sample limited to respondents working in hospital settings (*n* = 266). ^6^ Sample limited to respondents working in other care settings (*n* = 53).

**Table 2:** Factors associated with nurses’ knowledge of biosimilars.

		Level of Knowledge			
	Advanced	Intermediate	Basic	No Knowledge	Total
Hospital Unit					
ICU	0	3	11	17	31
Pharmacy	1	2	3	0	6
Ambulatory services	3	17	17	9	46
Inpatient	9	10	37	40	90
Other	13	13	37	30	93
Total	20	45	105	96	266
*V*					0.214 ^1^
Access to training					
No Access	9	43	152	141	345
Yes, delivered by pharmaceutical industry	13	14	9	0	36
Yes, delivered by my organization	1	3	6	4	14
Other	1	2	3	1	7
Total	24	62	170	146	402
*V*					0.284 ^1^
Use of Biosimilars					
No	2	1	21	13	37
Don’t know	0	3	32	73	108
Yes	22	58	117	60	257
Total	24	62	170	146	402
*V*					0.316 ^1^

^1^ *p* < 0.001. Outcomes between groups were compared using Fisher’s exact test. *V =* Cramer’s *V* coefficient, estimated to measure the strength of association (weak: >0.05; moderate: >0.10; strong: >0.15; and very strong: >0.25).

**Table 3:** Predictors for nurses’ knowledge of biosimilars.

Variable (Reference)	Predictor	Estimate	SE	Z	*p*	OR	95% CI Lower	95% CI Upper
Age (22–30)	31–40	0.390	0.476	0.818	0.413	1.476	0.5832	3.795
	41–50	0.421	0.437	0.964	0.335	1.524	0.6515	3.631
	51–65	0.484	0.459	1.055	0.292	1.623	0.6640	4.041
	≥66	−0.182	1.175	−0.155	0.877	0.834	0.0745	8.837
Hospital Unit (inpatient)	ICU	−0.3518	0.441	−0.797	0.425	0.703	0.2917	1.660
	Pharmacy	1.4932	0.789	1.893	0.058	4.451	0.9427	21.630
	Other	0.3285	0.319	1.029	0.303	1.389	0.7436	2.600
	Ambulatory	0.7598	0.373	2.037	0.042	2.138	1.0303	4.460
Use of Biosimilars (No)	Don’t know	−1.739	0.519	−3.348	<0.001	0.176	0.0626	0.482
	Yes	0.152	0.471	0.324	0.746	1.164	0.4617	2.940
Access to Training (No)	Yes, by organization	−0.3114	0.839	−0.371	0.711	0.732	0.1388	3.910
	Yes, by industry	2.4209	0.435	5.570	<0.001	11.256	4.8895	27.040
	Other	0.9368	0.771	1.215	0.225	2.552	0.5428	11.810

Model fit statistics: Deviance = 539; AIC = 571; McFadden’s R^2^ = 0.176. Estimate = regression coefficient; SE= standard error of the estimate; Z = Z-score of significance (high > 2); OR = odds ratio; CI = confidence interval. Note. Dependent variable: Knowledge (None | Basic | Intermediate | Advanced). Models estimated using *n* = 266.

**Table 4:** Predictors for nurses’ reliance on biosimilars.

Variable (Reference)	Predictor	Estimate	SE	Z	*p*	OR	95% CI Lower	95% CI Upper
Experience	(covariate)	0.0348	0.0268	1.297	0.194	1.035	0.98173	1.09
Age (22–30)	31–40	−0.0785	0.5402	−0.145	0.884	0.924	0.32059	2.68
	41–50	0.1227	0.6370	0.193	0.847	1.131	0.32658	3.99
	51–65	−0.6845	0.8779	−0.780	0.436	0.504	0.09103	2.87
	≥66	−19.061	14.581	−1.307	0.191	0.149	0.00802	2.63
Hospital Unit (Inpatient)	ICU	−0.1022	0.4372	−0.234	0.815	0.903	0.38013	2.12
	Pharmacy	0.6958	0.9275	0.750	0.453	2.005	0.35757	15.92
	Other	0.7642	0.3242	2.357	0.018	2.147	1.14169	4.08
	Ambulatory	0.9188	0.4013	2.290	0.022	2.506	1.15183	5.58
Knowledge (No)	Basic	1.3410	0.3121	4.296	<0.001	3.823	2.08957	7.12
	Intermediate	2.1412	0.4510	4.747	<0.001	8.510	3.58394	21.12
	Advanced	2.6806	0.6647	4.033	<0.001	14.594	4.30148	60.86
Use of Biosimilars (No)	Don’t know	−0.5364	0.5207	−1.030	0.303	0.585	0.20968	1.63
	Yes	13.761	0.4765	2.888	0.004	3.959	1.56337	10.21

Model fit statistics: Deviance = 434; AIC = 466; McFadden’s R^2^ = 0.238. OR = Odds Ratio. Note. Dependent variable: Confidence level (Low/None/Don’t know what a biosimilar is | Normal | High or total confidence). Models estimated using *n* = 266.

## Data Availability

The data that support the findings of this study are openly available in EUSurvey at https://ec.europa.eu/eusurvey/publication/biosimilares (accessed on 20 December 2025).

## Supplementary Materials

The following supporting information can be downloaded at: https://www.mdpi.com/article/10.3390/healthcare14040524/s1, Supplementary Table S1: Questions and categories of the survey tool. Some questions may have been skipped due to demographic characteristics of the participants or their responses to certain questions, Supplementary Table S2: Aggregation of categories to adjust effect bivariate and multivariate models, Supplementary Table S3: Nurses’ responses to knowledge, attitudes and perceptions toward biosimilars.

## Abbreviations

The following abbreviations are used in this manuscript:

ICU	Intensive Care Unit
OR	Odds Ratio
*r*_S_	Spearman’s rank correlation statistics (also known as *rHO*)
*C_V_*	Cramer’s *V* statistics
