# Supplementary material for: Assessing Nurses’ Knowledge and Attitudes Towards Biosimilars: Results from a National Survey
Source: Healthcare (Basel). 2026 Feb 19;14(4):524. doi: 10.3390/healthcare14040524 (PMC12940774; doi:10.3390/healthcare14040524)
Supplement: Supplementary file 1 [file healthcare-14-00524-s001.zip › Supp Table S1.pdf]

**Supplementary Table S1.** Questions and categories of the survey tool. Some questions may have been skipped due to demographic characteristics of the participants or their responses to certain questions.

| Category | Question | Answers |
|---|---|---|
| Demographic data | Which is your age group? | 22-30<br>31-40<br>41-50<br>51-65<br>>66 |
| Demographic data | Sex | Female<br>Male |
| Demographic data | In which Autonomous Community do you work? | Andalusia<br>Aragon<br>Balearic Islands<br>Canary Islands<br>Cantabria<br>Castilla la Mancha<br>Castilla y León<br>Catalonia<br>Community of Madrid<br>Autonomous Community of Navarre<br>Community of Valencia<br>Extremadura<br>Galicia<br>Basque Country<br>Principality of Asturias<br>Region of Murcia<br>La Rioja |
| Demographic data | What is your work experience in the profession? (years) | *45 years is the maximum value allowed* |
| Demographic data | What healthcare setting do you work in? | Primary Care<br>Hospital Care*<br>Other** |
| Demographic data | * What hospital unit do you work in? | Daycare Unit<br>Intensive Care Unit<br>Inpatient unit<br>Pharmacy Unit<br>Outpatient Clinics<br>Other |
| Demographic data | ** What other healthcare setting do you work in? | Social and health centre<br>Out-of-hospital emergencies<br>Mutual insurance/ occupational health companies<br>Other |
| Demographic data | The current work sector is: | Public<br>Private |

| | | |
|---|---|---|
| | | Semi-private |
| Demographic data | Do you have postgraduate training? Multiple response | Continuous training courses<br>Expert certificate<br>MSc.<br>PhD.<br>Nurse Specialist<br>No |
| Knowledge of biosimilars | My level of knowledge about biosimilars is: | None<br>Basic<br>Intermediate<br>Advanced |
| Knowledge of biosimilars | Which one of the following do you think best fits the definition of a biosimilar medicine? | It is a generic drug that has lost its patent and is equal to the original drug in efficacy and safety<br>It is a biological drug with a valid patent and can be manufactured by several pharmaceutical companies<br>It is a biological drug that has lost its patent and is equivalent in efficacy and safety to the original drug<br>It is a biological medicine similar to the original and whose efficacy and safety may not be equivalent to that of the original<br>None of the options seem right to me |
| Knowledge of biosimilars | Do you think it is the same to talk about a biosimilar as a generic? | Yes<br>No<br>I don't know |
| Knowledge of biosimilars | Which of the following biosimilars do you know? Multiple response | Adalimumab<br>Bevacizumab<br>Eculizumab<br>Enoxaparin sodium<br>Alpha and zeta poetins<br>Etanercept<br>Filgrastim<br>Follitropin alfa<br>Infliximab<br>Insulin glargine<br>Natalizumab<br>Pepfilgrastim<br>Ranibizumab<br>Rituximab<br>Somatropin<br>Teriparatide |

| | | |
|---|---|---|
| | | Tocilizumab<br>Trastuzumab<br>I don't know of any |
| Attitudes toward biosimilars | Would you like to receive training on biosimilars? | Yes<br>No |
| Attitudes toward biosimilars | What were the main sources of information, education or training you found about biosimilars? Multiple response | In the university career<br>Colleagues<br>In the workplace<br>Informative magazines<br>Guides<br>Congresses or Conferences<br>Training sessions<br>Courses<br>Social media<br>Internet sources<br>Scientific publications<br>I did not participate in any |
| Attitudes toward biosimilars | Can you tell if biosimilars are used in your workplace? | Yes<br>No<br>I don't know |
| Attitudes toward biosimilars | Do you have access to any courses on biosimilars? | Yes, delivered by my organization<br>Yes, delivered by the pharmaceutical industry<br>Other<br>No |
| Attitudes toward biosimilars | Do you think that the use of biosimilars could have any benefit in any of the following aspects? Multiple response | Reduce the cost of drugs<br>Greater sustainability<br>Better accessibility of drugs<br>It has benefit, but biosimilars can be an efficacy and safety risk for the patient<br>I don't know<br>Other |
| Attitudes toward biosimilars | How confident are you in the efficacy and safety of using biosimilar medicines? | I don't know what a biosimilar is<br>No trust<br>Low confidence<br>Normal<br>A lot of confidence<br>Total confidence |
| Attitudes toward biosimilars | What do you think your role should be as a nurse in health education in biosimilars? Multiple response | Information on the risk, benefit, quality, safety and efficacy of biosimilars<br>Information on its use, administration and the importance of adherence |

| | | |
|---|---|---|
| | | Management of routes of administration<br>Clarify doubts about biosimilars with the patient and, in some cases, with family members<br>I manage it, but I don't provide more information<br>I don't talk to the patient about biosimilars because I don't know much about it<br>Other |
| Attitudes toward biosimilars | Who do you think could provide information about biosimilars? Multiple response | Medical<br>Nurses<br>Pharmacists<br>The entire healthcare team that cares for the patient |
| Attitudes toward biosimilars | What do you think are the main barriers related to the use of biosimilars by healthcare workers? Multiple response | Lack of access in my workplace<br>Lack of sufficient knowledge<br>Lack of confidence in its use<br>Lack of experience in handling it<br>I don't know, I don't know |
| Attitudes toward biosimilars | How do you think the use of biosimilars by healthcare workers could be increased? Multiple response | Recommendations of Scientific Societies<br>Biosimilar Efficacy and Safety Information<br>Information on efficacy in interchangeability with the reference biologic<br>Other |
