# Supplementary material for: Assessing Nurses’ Knowledge and Attitudes Towards Biosimilars: Results from a National Survey
Source: Healthcare (Basel). 2026 Feb 19;14(4):524. doi: 10.3390/healthcare14040524 (PMC12940774; doi:10.3390/healthcare14040524)
Supplement: Supplementary file 1 [file healthcare-14-00524-s001.zip › Supp Table S2.pdf]

Supplementary Table S2. Aggregation of categories to adjust effect in bivariate and multivariate models.

Question	Original Categories	*n*	Aggregated categories	*n*
In which Autonomous Community do you work?	Andalusia	32	South	40
	Canary Islands	8		
	Principality of Asturias	5	North	137
	Aragon	36		
	Cantabria	2		
	Autonomous Community of Navarre	16		
	Castilla y León	44		
	Basque Country	15		
	Galicia	15		
	La Rioja	4		
	Community of Madrid	121	Midlands	142
	Extremadura	5		
	Castilla la Mancha	16		
	Catalonia	34	East	83
	Community of Valencia	11		
	Region of Murcia	36		
	Balearic Islands	2		
What hospital unit do you work in? *	Hospital Daycare Unit	21	Ambulatory Services	46
	Outpatient Clinics	25		
	Inpatient unit	90	Inpatient unit	90
	Pharmacy Unit	6	Pharmacy Unit	6
	Intensive Care Unit	31	Intensive Care Unit	31
	Other	93	Other	93
The current work sector is:	Public	336	Public	336
	Private	34	Private/Semi-private	66
	Semi-private	32		
Do you have postgraduate training? **	No	26	No certified training	124
	Continuing education courses	98		
	Expert certificate	80	Expert certificate	80
	MSc	93	MSc. /Nurse Specialist	189
	Nurse Specialist	96		
	PhD.	12	PhD.	12
How confident are you in the efficacy and safety of using biosimilar medicines?	I don't know what a biosimilar is	90	I don't know what a biosimilar is/No/Low confidence	103
	No trust	1		
	Low confidence	12		
	Average	151	Average	151
	Much confidence	83	Much or Total confidence	148
	Total confidence	65		

*Limited to individuals working in "hospital care" settings (*n*=266). ** Modified to academic degree achieved. *n* = group size.
