# Supplementary material for: Assessing Nurses’ Knowledge and Attitudes Towards Biosimilars: Results from a National Survey
Source: Healthcare (Basel). 2026 Feb 19;14(4):524. doi: 10.3390/healthcare14040524 (PMC12940774; doi:10.3390/healthcare14040524)
Supplement: Supplementary file 1 [file healthcare-14-00524-s001.zip › Supp Table S3.pdf]

**Supplementary Table S3.** Nurses' responses to knowledge, attitudes and perceptions toward biosimilars

| Questions and items | Frequency ($n$) | Rate |
|---|---|---|
| My level of knowledge about biosimilars is: | | |
| None | 146 | 36.32 % |
| Basic | 170 | 42.29 % |
| Intermediate | 62 | 15.42 % |
| Advanced | 24 | 5.97 % |
| Which one of the following do you think best fits the definition of a biosimilar medicine? | | |
| It is a generic drug that has lost its patent and is equal to the original drug in efficacy and safety | 34 | 8.46 % |
| It is a biological drug with a valid patent and can be manufactured by several pharmaceutical companies | 75 | 18.66 % |
| It is a biological drug that has lost its patent and is equivalent in efficacy and safety to the original drug | 187 | 46.52 % |
| It is a biological medicine similar to the original and whose efficacy and safety may not be equivalent to that of the original | 73 | 18.16 % |
| None of the options seem right to me | 33 | 8.21 % |
| Do you think it is the same to talk about a biosimilar as a generic? | | |
| Yes | 43 | 10.7 % |
| No | 269 | 66.92 % |
| I don't know | 90 | 22.39 % |
| Which of the following biosimilars do you know? Multiple response | | |
| Adalimumab | 119 | 29.6 % |
| Bevacizumab | 50 | 12.44 % |
| Eculizumab | 40 | 9.95 % |
| Enoxaparin sodium | 226 | 56.22 % |
| Alpha and zeta poetins | 66 | 16.42 % |
| Etanercept | 56 | 13.93 % |
| Filgrastim | 86 | 21.39 % |
| Follitropin alfa | 10 | 2.49 % |
| Infliximab | 132 | 32.84 % |
| Insulin glargine | 195 | 48.51 % |
| Natalizumab | 37 | 9.2 % |
| Pepfilgrastim | 17 | 4.23 % |
| Ranibizumab | 37 | 9.2 % |
| Rituximab | 146 | 36.32 % |
| Somatropin | 93 | 23.13 % |
| Teriparatide | 40 | 9.95 % |
| Tocilizumab | 105 | 26.12 % |
| Trastuzumab | 47 | 11.69 % |
| I don't know of any | 73 | 18.16 % |
| Would you like to receive training on biosimilars? | | |
| Yes | 359 | 89.3 % |
| No | 43 | 10.7 % |

| | | |
|---|---|---|
| What were the main sources of information, education or training you found about biosimilars? Multiple response | | |
| In the university career | 17 | 4.23 % |
| Colleagues | 79 | 19.65 % |
| In the workplace | 176 | 43.78 % |
| Informative magazines | 22 | 5.47 % |
| Guides | 40 | 9.95 % |
| Congresses or Conferences | 57 | 14.18 % |
| Training sessions | 55 | 13.68 % |
| Courses | 42 | 10.45 % |
| Social media | 33 | 8.21 % |
| Internet sources | 72 | 17.91 % |
| Scientific publications | 50 | 12.44 % |
| I did not participate in any | 128 | 31.84 % |
| Can you tell if biosimilars are used in your workplace? | | |
| Yes | 257 | 63.93 % |
| No | 37 | 9.2 % |
| I don't know | 108 | 26.87 % |
| Do you have access to any courses on biosimilars? | | |
| Yes, delivered by my organization | 14 | 3.48 % |
| Yes, delivered by the pharmaceutical industry | 36 | 8.96 % |
| Other | 7 | 1.74 % |
| No | 345 | 85.82 % |
| Do you think that the use of biosimilars could have any benefit in any of the following aspects? Multiple response | | |
| Reduce the cost of drugs | 213 | 52.99 % |
| Greater sustainability | 130 | 32.34 % |
| Better accessibility of drugs | 171 | 42.54 % |
| It has benefit, but biosimilars can be an efficacy and safety risk for the patient | 32 | 7.96 % |
| I don't know | 136 | 33.83 % |
| Other | 1 | 0.25 % |
| How confident are you in the efficacy and safety of using biosimilar medicines? | | |
| No confidence at all | 1 | 0.25 % |
| Low confidence | 12 | 2.99 % |
| Normal | 151 | 37.56 % |
| A lot of confidence | 83 | 20.65 % |
| Total confidence | 65 | 16.17 % |
| I don't know what a biosimilar is | 90 | 22.39 % |
| What do you think your role should be as a nurse in health education in biosimilars? Multiple response | | |
| Information on the risk, benefit, quality, safety and efficacy of biosimilars | 236 | 58.71 % |
| Information on its use, administration and the importance of adherence | 273 | 67.91 % |
| Management of routes of administration | 246 | 61.19 % |
| Clarify doubts about biosimilars with the patient and, in some cases, with family members | 247 | 61.44 % |
| I manage it, but I don't provide more information | 14 | 3.48 % |

| | | |
|---|---|---|
| I don't talk to the patient about biosimilars because I don't know much about it | 82 | 20.4 % |
| Other | 2 | 0.5 % |
| Who do you think could provide information about biosimilars? Multiple response | | |
| Medical | 169 | 42.04 % |
| Nurses | 170 | 42.29 % |
| Pharmacists | 186 | 46.27 % |
| The entire healthcare team that cares for the patient | 263 | 65.42 % |
| What do you think are the main barriers related to the use of biosimilars by healthcare workers? Multiple response | | |
| Lack of access in my workplace | 60 | 14.93 % |
| Lack of sufficient knowledge | 276 | 68.66 % |
| Lack of confidence in its use | 71 | 17.66 % |
| Lack of experience in handling it | 150 | 37.31 % |
| I don't know | 91 | 22.64 % |
| How do you think the use of biosimilars by healthcare workers could be increased? Multiple response | | |
| Recommendations of Scientific Societies | 219 | 54.48 % |
| Biosimilar Efficacy and Safety Information | 311 | 77.36 % |
| Information on efficacy in interchangeability with the reference biologic | 263 | 65.42 % |
| Other | 14 | 3.48 % |
